# Heterogeneous Responses of Ovarian Cancer Cells to Silver Nanoparticles as a Single Agent and in Combination with Cisplatin

**DOI:** 10.1155/2017/5107485

**Published:** 2017-04-26

**Authors:** Cale D. Fahrenholtz, Jessica Swanner, Maria Ramirez-Perez, Ravi N. Singh

**Affiliations:** 1 Department of Cancer Biology, Wake Forest School of Medicine, Winston-Salem, NC 27157, USA; 2 Salem College, Winston-Salem, NC 27101, USA; 3 Comprehensive Cancer Center of Wake Forest Baptist Medical Center, Winston-Salem, NC 27157, USA

## Abstract

We investigated the effects of silver nanoparticle (AgNP) exposure in three ovarian cancer cell lines (A2780, SKOV3, and OVCAR3). We found that AgNPs were highly cytotoxic toward A2780 and SKOV3 cells but OVCAR3 cells were less sensitive to AgNPs. In agreement with the cytotoxicity data, AgNPs caused DNA damage in A2780 and SKOV3 cells, but not in OVCAR3 cells. A2780 and SKOV3 showed higher levels of basal reactive oxygen species (ROS) relative to OVCAR3 cells. AgNP exposure increased ROS levels in both A2780 and SKOV3 cells, but not in OVCAR3 cells. We found that the heterogeneous cytotoxicity was specific to the uptake of intact particles and was not due to differences in sensitivity to silver ions. Furthermore, the combination of AgNPs and standard-of-care platinum therapy, cisplatin (*cis*-diamminedichloroplatinum(II), CDDP), was synergistic for treatment of A2780 andOVCAR3 cells and the combination of AgNPs and CDDP showed a favorable dose reduction in all cell lines tested. These results provide insight into potential applications of AgNPs for treatment of ovarian cancer.

## 1. Introduction

Ovarian cancer is the most lethal of all gynecological malignancies with a five-year survival rate of only 40% and is the fourth leading cause of female cancer deaths in the United States [1]. Over 80% of ovarian cancers are classified as high-grade serous carcinoma which frequently has defects in pathways involved in DNA damage responses [2]. DNA damaging drugs including cisplatin (CDDP) are among the most effective agents available to clinicians for treatment of ovarian cancer [3], but this efficacy comes at the expense of significant dose-limiting side effects [4]. Advances in nanotechnology, the application of materials in the size range between 1 and 100 nm in dimension, may enable the development of more effective and less toxic cancer treatments [5, 6].

Among nanomaterials, silver nanoparticles (AgNPs) are already well established in human medicine. The clinical safety and efficacy of AgNPs have been demonstrated for application in wound dressings [7], bactericides [8–10], and coatings for implantable medical devices [11]. Preclinical studies on AgNPs show that they possess a cytotoxic activity toward a variety of cancer cell lines and in animal models of cancer following intratumoral injection [12–22]. AgNP exposure can lead to dose dependent apoptotic and necrotic cell death, in part due to DNA damage and induction of oxidative stress [23, 24]. We found that AgNPs are highly cytotoxic to aggressive, triple-negative breast cancer cells at doses that had no effect on noncancerous breast cells and cells derived from the liver, kidney, or macrophages [25], indicating that a therapeutic window exists for the safe use of AgNPs. Significantly, cells deficient in their capacity to repair DNA damage may be more susceptible to AgNP toxicity [24].

As AgNPs have been shown to enhance the efficacy of chemotherapeutic drugs including 5-fluorouracil [26] and doxorubicin [27], AgNPs could be useful in conjunction with standard-of-care platinum therapy. Furthermore, because AgNPs exposure depletes cells of reduced glutathione [28], a thiol antioxidant associated with cisplatin resistance [29], the combination of AgNPs and cisplatin could be particularly effective. Thus far, combined effects of AgNPs with cisplatin have not been assessed. Moreover, it remains unknown whether a synergistic or dose reducing interaction between AgNPs and cisplatin exists.

In this report, we evaluate the efficacy of AgNPs for treatment of SKOV3, A2870, and OVCAR3 ovarian cancer cells, which are commonly used as models of high-grade serous carcinoma [30]. We quantify cell viability, assess glutathione levels, and monitor reactive oxygen species (ROS) and also DNA damage after AgNP treatment. Because platinum-based chemotherapy remains a first-line choice for treatment of ovarian cancer, we subsequently determined the effect of combination therapy using both AgNPs and cisplatin to treat these cell lines. Our results provide insight into potential applications of AgNPs for treatment of ovarian cancer.

## 2. Experimental Section

### 2.1. Cell Lines and Reagents

A2780, SKOV3, and OVCAR3 ovarian cancer cells were purchased from ATCC. All cells were maintained in RPMI (Lonza) supplemented with 10% fetal bovine serum (FBS) (Sigma-Aldrich), 100 IU/ml penicillin (Life Technologies), and 100 *μ*g/ml streptomycin (Life Technologies). Cisplatin (CDDP) was obtained from Cayman Chemicals. Cytochalasin D was obtained from Sigma-Aldrich.

### 2.2. Silver Nanoparticles

A powder of 25nm AgNPs capped with polyvinylpyrrolidone (PVP) (Ag : PVP 15 : 85) with a mean diameter of 23.1 ± 6.9 nm (assessed by transmission electron microscopy) was obtained from nanoComposix. AgNPs were dispersed in phosphate-buffered saline (PBS) (Lonza) at a concentration of 20mg/ml and briefly sonicated, and the stock suspension was stored at 4°C in the dark for no longer than one month.

### 2.3. Dynamic Light Scattering (DLS)

AgNPs were diluted to ~40 *μ*g/ml in water or PBS. Hydrodynamic diameter and zeta-potential were assessed using a ZetaSizer Nano ZS90 (Malvern). Hydrodynamic diameter was measured in deionized water and PBS and zeta-potential was determined in deionized water. Each measurement was performed in triplicate at 25°C.

### 2.4. Transmission Electron Microscopy (TEM)

AgNPs in deionized water were pipetted on copper coated formvar grids and allowed to dry. Grids were imaged using a Tecnai Spirit transmission electron microscope.

### 2.5. MTT Assays for Cytotoxicity and Synergy Studies

4–5 × 10^3^ ovarian cancer cells were plated on 96-well tissue culture plates (BD Falcon) and allowed to attach overnight. Cells were treated as indicated. Medium was removed using gentle aspiration and replaced with fresh growth medium containing thiazolyl blue tetrazolium bromide (0.5mg/ml). Plates were incubated at 37°C for 30–90 minutes, and medium was replaced with dimethyl sulfoxide. Wells were mixed using a micropipette and absorbance was read at 560 nm and corrected using a reference wavelength of 650 nm using a Molecular Devices Emax Precision Microplate Reader. Synergy and dose reduction index analysis was performed using CompuSyn software version 1.0.

### 2.6. Reactive Oxygen Species (ROS) Microscopy

A2780, SKOV3, and OVCAR3 cells (0.5–1.0 × 10^5^ cells) were seeded on 24-well tissue culture plates and allowed to attach overnight. On the following day, cells were treated as indicated for 24 hours at 37°C. Medium was removed, and cells were washed with PBS (with magnesium and calcium) and incubated with 10 *μ*M 2′,7′-dichlorodihydrofluorescein diacetate (H_2_DCF-DA) (Invitrogen) diluted in PBS (with calcium and magnesium) for 5 min at 37°C. Cells were imaged using EVOS FL Auto (Thermo Scientific).

### 2.7. Enzyme-Linked Immunosorbent Assay

A2780, SKOV3, and OVCAR3 (1.0–1.5 × 10^4^ cells) were seeded on black-walled 96-well tissue culture plates and allowed to attach overnight. On the following day, cells were treated as indicated for 24 h. Levels of phosphorylated H2AX (S139) and total H2AX were assessed by enzyme-linked immunosorbent assay (ELISA) according to the manufacturer’s protocol (R&D Systems) using a Molecular Devices FMax Precision Microplate Reader with excitation/emission filter pairs set to 360 nm/450 nm and 540 nm/600 nm, respectively.

### 2.8. Glutathione Assay

A2780, SKOV3, and OVCAR3 (1.0–1.5 × 10^4^ cells) were seeded on white-walled 96-well tissue culture plates and allowed to attached overnight. On the following day, cells were treated as indicated for 24 h. Levels of oxidized glutathione and total glutathione were determined using a GSH/GSSG Glo Assay (Promega) according to the manufacturer’s instructions.

## 3. Results

### 3.1. Ovarian Cancer Cell Lines Exhibit Heterogeneous Sensitivities to AgNP Exposure

**Figure 1:**
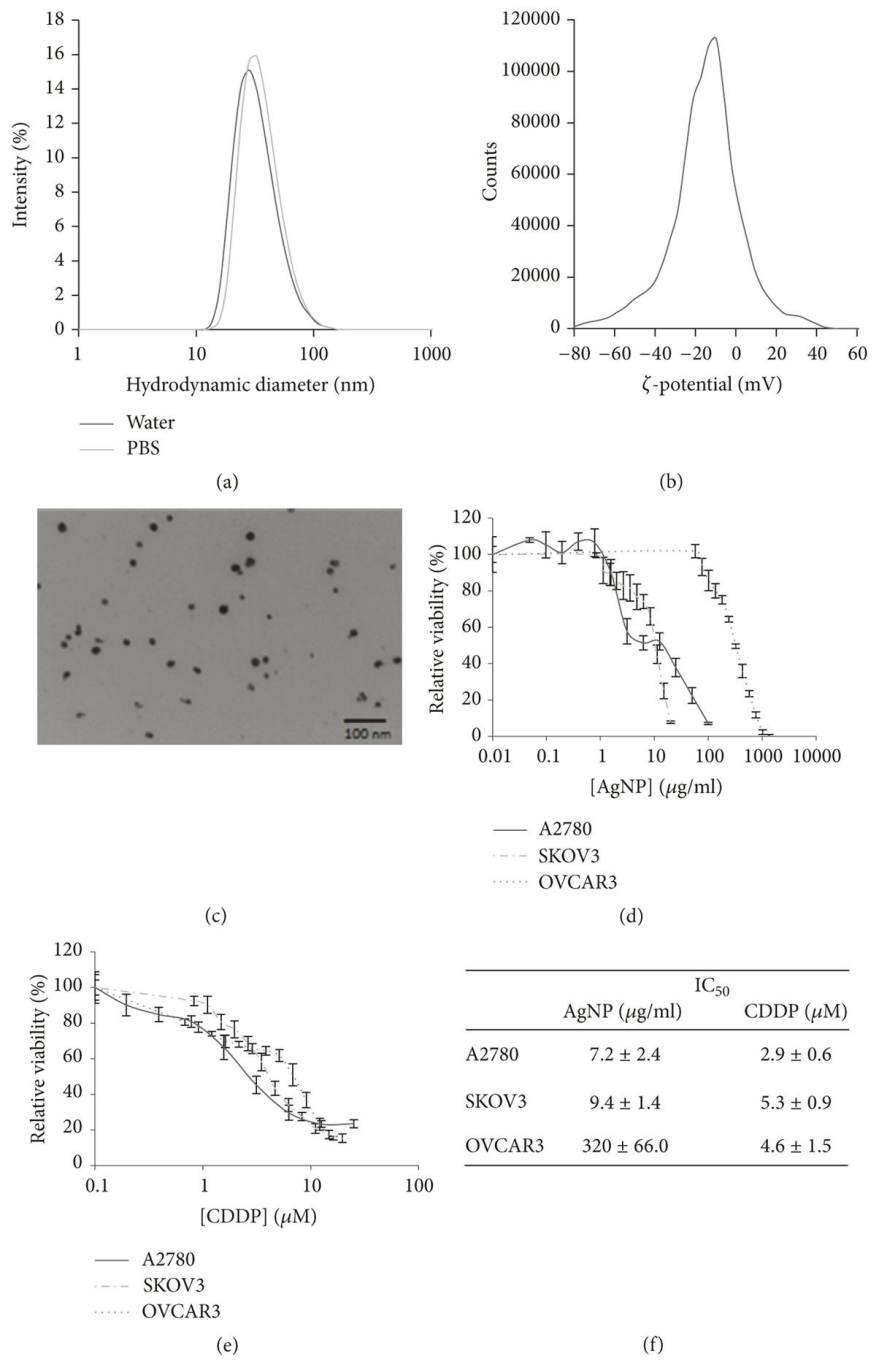
Physicochemical characterization and cytotoxicity of AgNPs PVP-coated silver nanoparticles were dispersed in water or PBS (40 *μ*g/ml), briefly sonicated, and incubated at room temperature for 1 hour. (a)Hydrodynamic diameter for AgNPs in water or PBS is shown. (b) ζ-potential for AgNPs dispersed in water is shown. (c) Electron micrographs of AgNPs (scale bar = 100 nm) are shown. A2780, SKOV3, and OVCAR3 cells were seeded and allowed to attach overnight and then treated with (d) AgNP (0–1000 *μ*g/ml) or (e) CDDP (0–25 *μ*M) for 72 hours. Cell viability was assessed by MTT assay. Data is shown as percent viability compared to vehicle treated cells. (f) IC_50_ values were determined from three independent experiments. All data is shown ± standard deviation.

Spherical silver nanoparticles coated with the biocompatible polymer polyvinylpolypyrrolidone (PVP) were used for these studies. They possess a Ag : PVP mass ratio of 15 : 85 and nominal diameter of 23.1nm according to the supplied manufacturer’s data sheet (Supplementary Table 1 in Supplementary Material available online at https://doi.org/10.1155/2017/5107485). We verified the particle hydrodynamic diameter in water (24.1 ± 0.4 nm) and PBS (23.5 ± 0.5 nm) using dynamic light scattering (Figure 1(a)). There was no evidence of AgNP agglomeration (no increase in hydrodynamic diameter) in water or PBS over time, and no sedimentation was observed. Zeta-potential in water was determined to be −14.8 ± 0.5mV (Figure 1(b)). After AgNPs were hydrated and then dried on copper coated formvar grids, imaging by transmission electron microscopy (TEM) indicated that the particles remained individualized (Figure 1(c)).

We treated three ovarian cancer cell lines (A2780, SKOV3, and OVCAR3) with AgNPs at concentrations ranging from 0 to 1000 *μ*g/ml. AgNPs were highly cytotoxic to both A2780 and SKOV3 after 72 h treatment but were less cytotoxic to OVCAR3 cells (Figure 1(d)). In contrast to the differences in sensitivity to AgNP treatment, all cell lines tested were equally sensitive to CDDP (Figure 1(e)). IC_50_ values were determined for both AgNP and CDDP after 72 h treatment (Figure 1(f)).

### 3.2. Uptake of Intact AgNPs Is Necessary for Differences in AgNP Cytotoxicity among Ovarian Cancer Cell Lines

**Figure 2:**
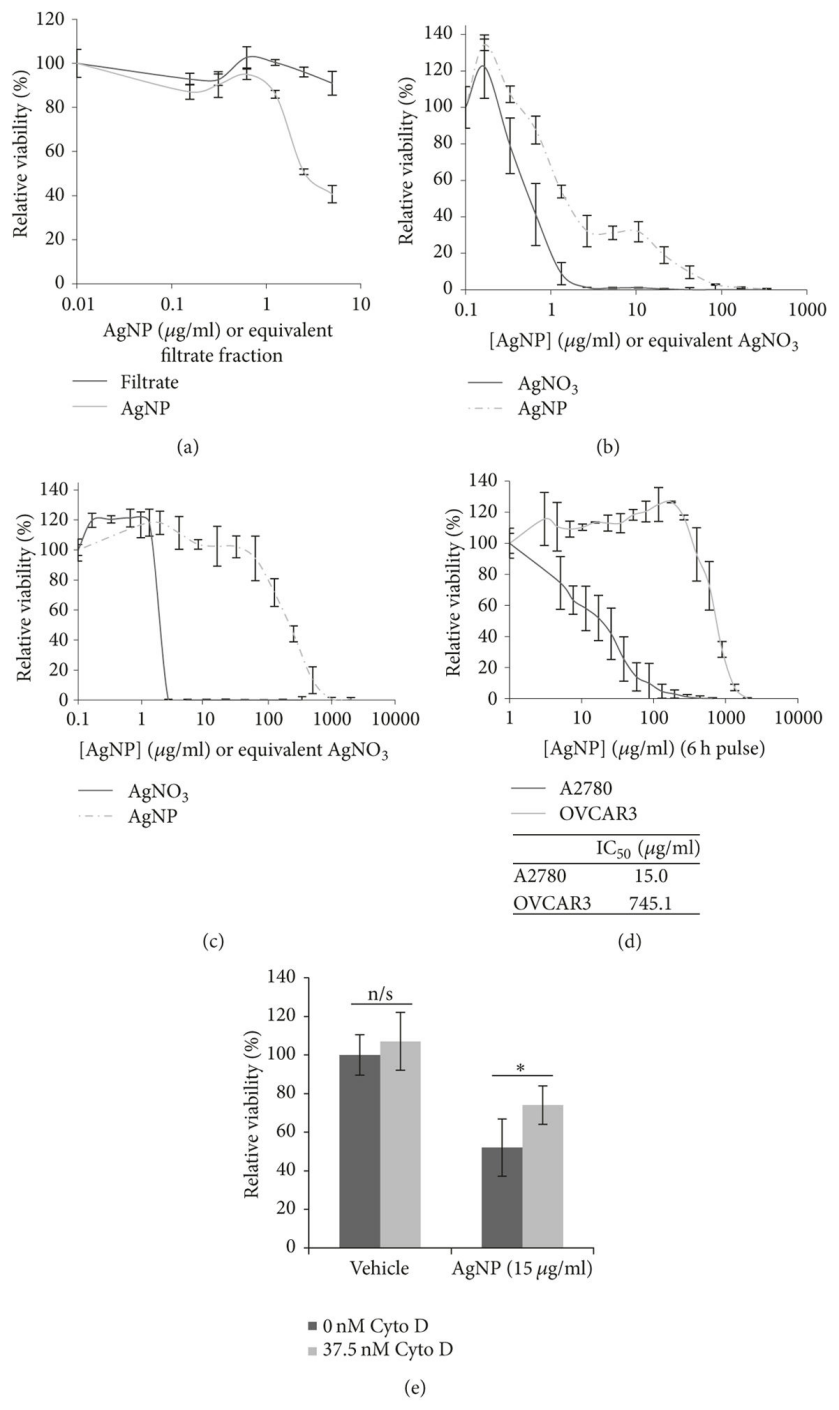
Cell line differences in cytotoxicity after AgNP treatment are specific to exposure to and uptake of intact nanoparticles (a) A2780 cells were seeded and allowed to attach overnight and then treated for 72h with AgNPs or an equivalent volume of filtrate separated from AgNPs. (b) A2780 or (c) OVCAR3 cells were seeded and allowed to attach overnight and then treated with AgNP or AgNO_3_ (dosed by mass of Ag) for 72h. Viability was assessed by MTT assay and all data is shown as percent viability relative to vehicle treated cells ± standard deviation. (d) A2780 and OVCAR3 cells were seeded and allowed to attach overnight and then treated with AgNP (0–2000 *μ*g/ml) for 6 h. Treatment medium was replaced with normal growth medium and the cells were allowed to recover for 72 h. Calculated IC_50_ values are shown in the inset. (e) A2780 were treated as above with AgNP (0, 15 *μ*g/ml) and cytochalasin D (cyto D, 37.5 nM) included in the 6-hour treatment. Cell viability was assessed by MTT assay and data is shown as percent viability relative to vehicle treated cells ± standard deviation. Significant differences are indicated: ^*^p < 0.05; Student’s *t*-test.

Next, we determined whether degradation products potentially released from AgNPs during storage contributed to the cytotoxicity. After storage of AgNPs in water for 30 days, intact nanoparticles were separated from dissolved components (potentially including silver ions (Ag+)) by filtration through a centrifugal size exclusion column. Dilutions of each fraction (filtrate or particle) were prepared based upon the initial concentration of AgNPs added to the column. A2780 cells, which were the most sensitive cell line to AgNPs, were treated with increasing doses of each fraction to assess cytotoxicity. In agreement with our previous studies [25], we found that the AgNP-mediated cytotoxicity was dependent upon exposure of cells to intact AgNPs and not due to substances released during processing or storage of the nanoparticles (Figure 2(a)). To determine whether differences in cell line sensitivity/tolerance to Ag+ could play a role in the relative efficacy of AgNPs for treatment of ovarian cancer cells, we exposed OVCAR3 and A2780 cells, the cell lines that were least and most sensitive to AgNP exposure, respectively, to intact AgNPs or an equivalent molar concentration of Ag+ (using AgNO_3_ as the ion source). In contrast to differences in sensitivity to AgNP exposure, both cell lines exhibited similar sensitivity to Ag+, providing further evidence that the heterogeneous responses to AgNPs among the cell lines were dependent upon exposure to the intact nanoparticles (Figures 2(b) and 2(c)).

Because our data showed that the cytotoxic effects of AgNPs were due to intact nanoparticles, we developed studies to investigate whether cytotoxicity was dependent on uptake of AgNPs. Cytochalasin D (cyto D) is a cell-permeable actin depolymerizing agent that inhibits endocytosis, but extended exposure to cyto D is cytotoxic. All of our previous studies involved continuous exposure of cells to AgNPs for 72 h, which is a too long time period during which to use cyto D. Therefore, we tested to see whether cells could be treated with AgNPs for a shorter time period but still achieve similar cytotoxicity. A2780 and OVCAR3 cells were treated with AgNP at concentrations ranging from 0 to 2000 *μ*g/ml for 6 hours. AgNPs were then removed and cells were allowed to recover for 66 h in normal growth medium. We found that even acute exposure (6 h pulse; 66 h recovery) showed cytotoxic effects similar to chronic (continuous exposure to AgNPs for 72 h) treatment (Figure 2(d)). Calculated IC_50_ values of acute AgNP exposure were approximately 2-fold greater than of chronic exposure (7.2 versus 15.0 *μ*g/ml for A2780 and 320.0 versus 745.1 *μ*g/ml for OVCAR3, acute versus chronic, resp.).

Having determined that exposure to AgNPs for 6 h was sufficient to induce cytotoxicity in A2780 cells, we next determined whether endocytosis was involved in AgNP-mediated cytotoxicity. We pulsed A2780 cells for 6 h with AgNPs (at the IC_50_ dose determined above) in the presence of a nontoxic dose of cyto D (37.5 nM) and then replaced the nanoparticle/drug containing media with fresh media and allowed cells to recover for 66 h. In accordance with our previous studies [25], we found that the addition of cyto D abrogated a significant portion of AgNP-induced cytotoxicity (Figure 2(e)).

### 3.3. Sensitivity to AgNPs among Ovarian Cancer Cell Lines Is Correlated with Basal Levels of ROS and Induction of DNA Damage

**Figure 3:**
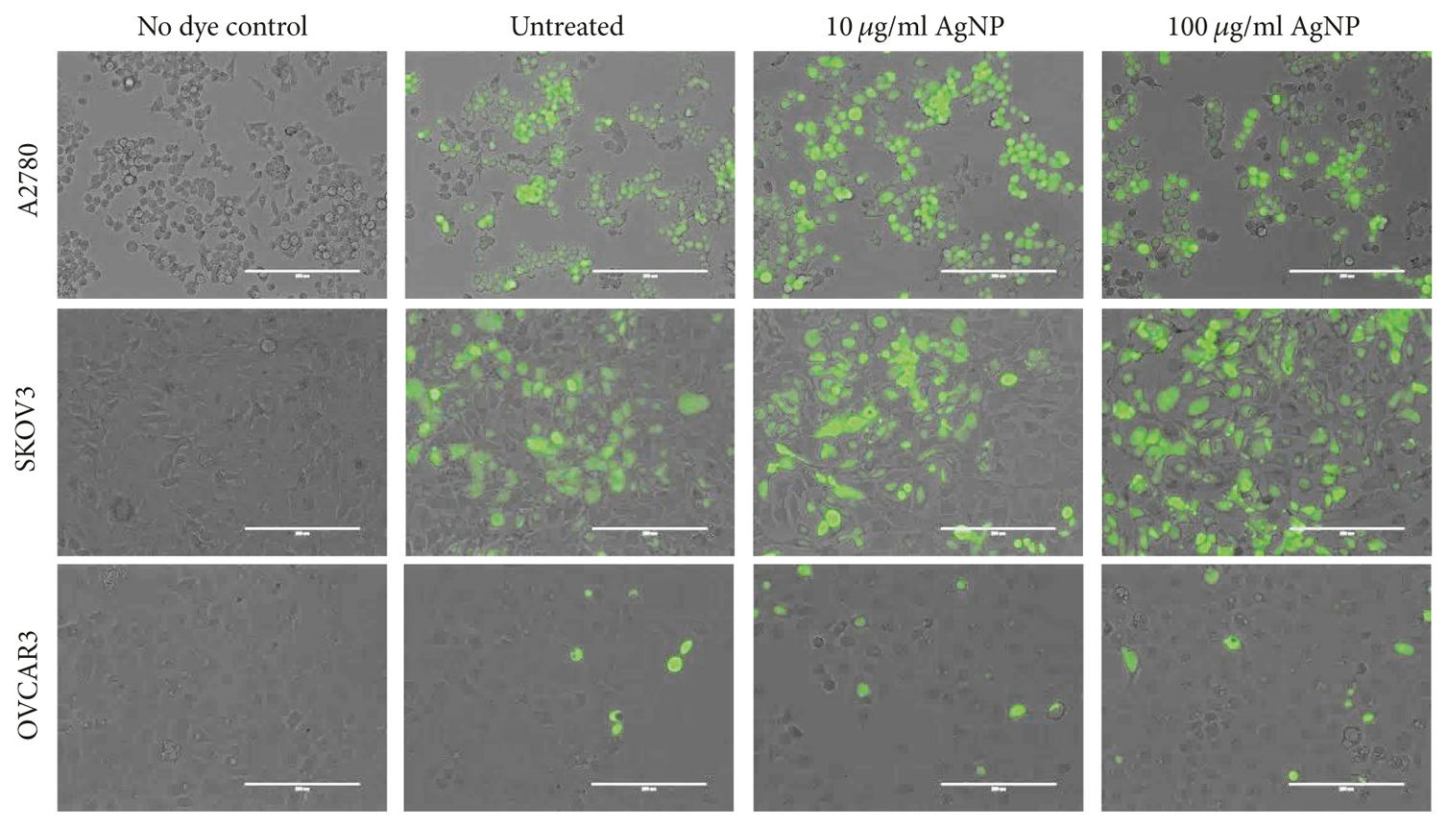
Quantification of ROS in ovarian cancer cells before and after NP exposure A2780, SKOV3, and OVCAR3 cells were seeded and allowed to attach overnight and then treated with AgNP (10 or 100*μ*g/ml) or vehicle for 24 hours. Cells were washed with PBS and incubated with PBS containing the ROS responsive dye, H_2_DCF-DA, and then ROS was assessed by fluorescence microscopy. Dye-free controls of cells treated were used to verify the specificity of the fluorescence for ROS detection.

AgNP exposure may increase intracellular ROS levels [22]. Oxidative damage caused by increased ROS is thought to contribute to AgNP-mediated cell death. Therefore, we assessed intracellular ROS levels using a ROS responsive fluorogenic probe. We found that the AgNP-sensitive cell lines A2780 and SKOV3 both had higher baseline levels of ROS compared to the AgNP-insensitive OVCAR3 cells (Figure 3). Following 24 h exposure to 10 *μ*g/ml AgNP doses, ROS levels increased relative to baseline in both of the AgNP-sensitive cell lines (A2780 and SKOV3). At a higher AgNP dose (100 *μ*g/ml), ROS levels increased in SKOV3 relative to baseline, but this was not apparent in A2780 cells treated at the same dose. However, of the three cell lines tested, A2780 cells were the most sensitive to AgNP exposure. After these cells were treated with a 100 *μ*g/ml dose of AgNPs, many cells became rounded and there was a significant loss of adherent cells. Therefore, it is likely that loss of cell viability contributed to the lack of a dose dependent increase in ROS in A2780 following AgNP exposure. However, AgNPs did not alter ROS levels in less sensitive OVCAR3 cells at any tested concentration.

Glutathione (GSH) is a ubiquitous tripeptide antioxidant that plays a key role in mitigating oxidative damage. GSH is oxidized by ROS to form a homodimer disulfide (GSSG). AgNPs were shown to decrease the GSH/GSSG ratio in some cells [28], but this had not been assessed in ovarian cancer cells. The ratio between GSH and GSSG can be used as a metric to define the redox state of a cell [31], and imbalances in this ratio leading to excess GSSG can cause cell death [32]. Therefore, we treated A2780, SKOV3, and OVCAR3 cells with AgNPs (0, 10, and 100 *μ*g/ml) for 24 h and then quantified the cellular content of both oxidized (GSSG) and reduced glutathione (GSH). This time point was selected because, after 24 h exposure to AgNPs, significantly less cytotoxicity was observed compared to 72 h (Supplementary Figure S1a; Figure 1), which enabled quantification of sublethal effects of AgNPs that occur prior to cell death. In parallel, treatment of cells with glutathione synthesis inhibitor, buthionine sulfoximine (BSO), was used as a positive control for GSH depletion (Supplementary Figure S1b).

**Figure 4:**
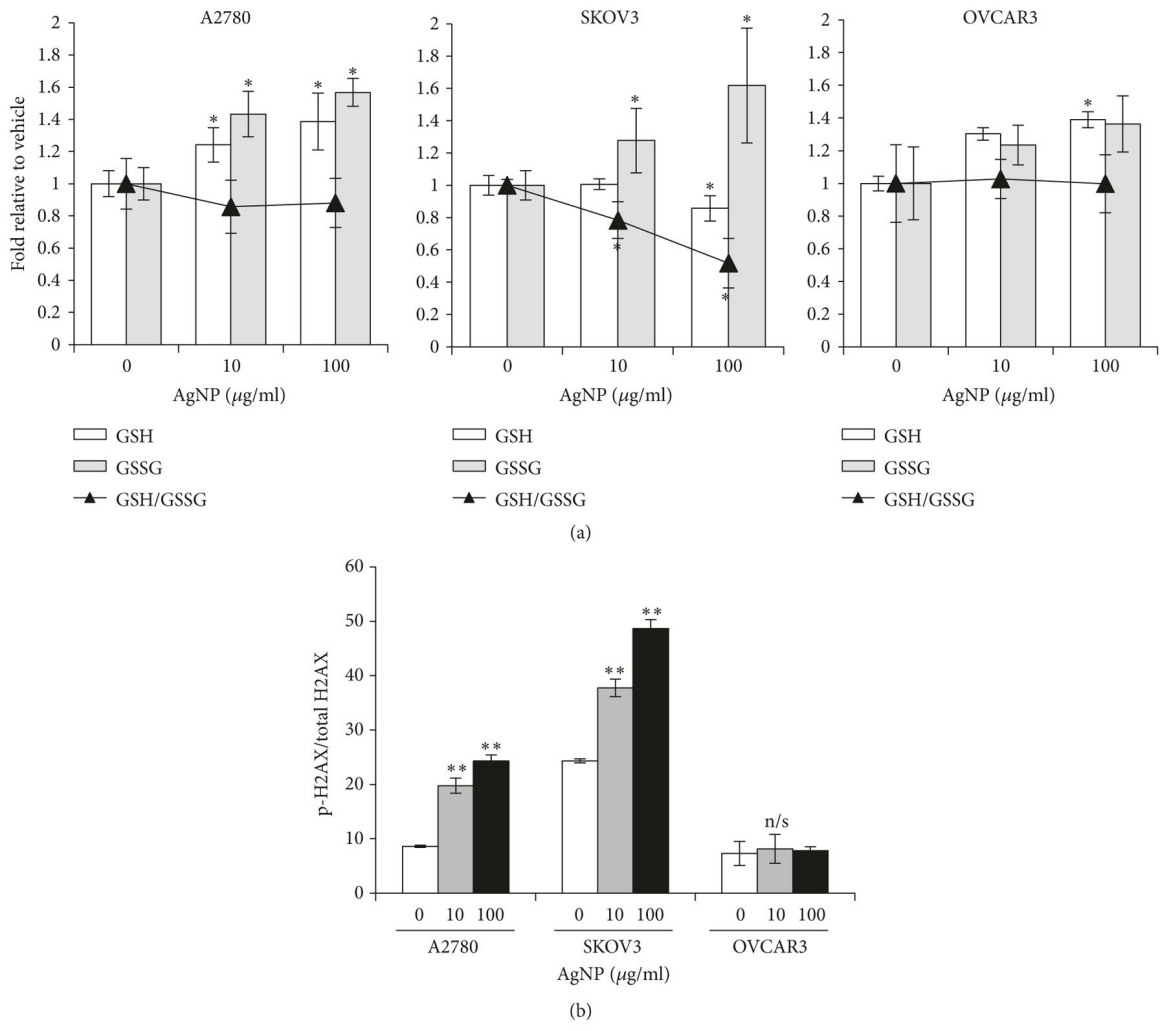
Quantification of glutathione and DNA damage in ovarian cancer cells before and after AgNP exposure A2780, SKOV3, andOVCAR3 cells were seeded and allowed to attach overnight and then treated with AgNP (10 or 100 *μ*g/ml) or vehicle for 24 hours. (a) Cells were assessed for reduced GSH and oxidized GSSG. Data are shown as fold relative to vehicle treated control for each cell line with the GSH/GSSG ratio ± SD in laid. (b) Phosphorylated (S139) and total H2AX were determined by ELISA. Data is shown as phospho/total *γ*-H2AX relative to vehicle treatment ± standard deviation for individual cell lines. Significant differences are indicated: ^*^p < 0.05; ^**^p < 0.01; Student’s *t*-test. through both synergistic interactions and dose reducing capabilities.

After AgNP treatment, a dose dependent increase in GSSG was observed in both A2780 and SKOV3 cells, but not in OVCAR3 cells (Figure 4(a)). GSH increased in A2780 and in OVCAR3 cells, but a dose dependent decrease in GSH was observed in SKOV3 cells. The net effect of these changes was a decrease in the GSH/GSSG ratio in SKOV3 cells, but not in OVCAR3 and A2780 cells. The lack of correlation between the effects of AgNPs on GSH/GSSG and relative sensitivity of ovarian cancer cells to AgNP exposure indicated that modulation of the GSH/GSSG ratio is unlikely to be the dominant mechanism by which AgNPs exert their cytotoxic effects.

Next, we quantified phosphorylated H2AX, one of the earliest detectable indicators of double strand DNA breaks [33]. A2780, SKOV3, and OVCAR3 cells were treated with AgNPs under the same conditions as above, and the amount of phosphorylated H2AX was normalized to total H2AX. The AgNP treatment induced significant DNA damage in A2780 and SKOV3 (Figure 4(b)). In contrast, little indication of DNA double strand breaks was observed following treatment of OVCAR3 with AgNPs. These data mirror our cytotoxicity data and indicate that induction of DNA damage likely contributes to the sensitivity of A2780 and SKOV3 to AgNPs.

### 3.4. The Combination of AgNPs and Cisplatin Shows Synergism and a Favorable Dose Reduction for Treatment of Ovarian Cancer Cells

Platinum-based therapy, including cisplatin (CDDP), is a mainstay for standard-of-care therapy for ovarian cancer [3]. Therefore, we investigated whether the combination of AgNP and CDDP resulted in a synergistic, additive, or antagonistic interaction in treatment efficacy compared to the individual agents using the Chou-Talalay method for drug combination analysis [34]. This method of analysis requires that cells be treated with a combination of AgNPs and CDDP at a fixed drug ratio. For determination of synergy, cells should be treated with increasing doses of the drug combination that are fractions or multiples of a constant dose ratio of IC_50_ for the individual agents. Based upon triplicate experiments (similar to those shown in Figure 1), these ratios (AgNP (*μ*g/ml) : CDDP (*μ*M)) were defined individually for each cell line as 2.5 : 1 for A2780; 63.2 : 1 for OVCAR3; and 1.5 : 1 for SKOV3.

**Figure 5:**
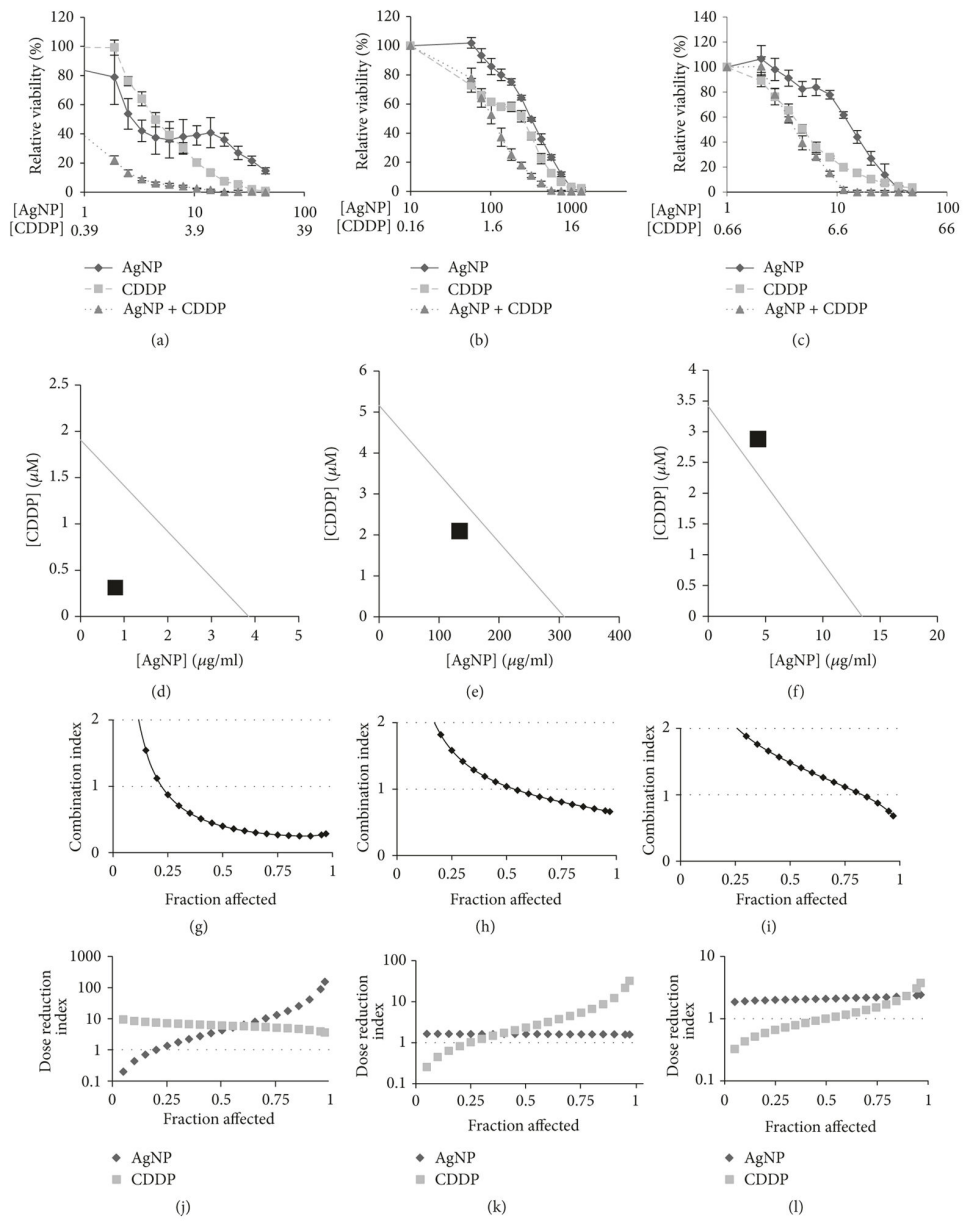
AgNPs synergize with cisplatin and show favorable dose reduction (a) A2780, (b) OVCAR3, and (c) SKOV3 cells were treated with AgNP and cisplatin (CDDP) alone or in combination at constant ratios based on IC_50_ (AgNP (*μ*g/ml) : CDDP (*μ*M), 7.9 : 3.1 for A2780; 322.3 : 5.1 for OVCAR3; and 5.7 : 8.6 for SKOV3) for 72 hours in normal growth medium. Cell viability was assessed by MTT assay and is shown as percent viability relative to vehicle ± standard deviation for AgNP alone, CDDP alone, and the combination of AgNP and CDDP. Additive isoboles (*F*_a_ = 0.5) are shown for (d) A2780, (e) OVCAR3, and (f) SKOV3. Plots indicating the combinatorial index for AgNP and CDDP are shown for (g) A2780, (h) OVCAR3, and (i) SKOV3. Dose reduction index is also shown for (j) A2780, (k) OVCAR3, and (l) SKOV3. Synergy analysis (including combinatorial index and dose reduction index) was calculated using CompuSyn software.

Next, studies were performed to assess synergy of the combination of AgNPs and CDDP. The three ovarian cancer cell lines were exposed to each agent alone or increasing concentrations of the combined treatment with the ratio between the two agents remaining constant as defined above. Cell viability was quantified 72 h later by MTT assay as shown (Figures 5(a)–5(c)). For analysis, the additive isobole was plotted for each cell line at a fraction affected (*F*_a_) of 0.5 (indicating 50% loss of cell viability). In the additive isobole, synergy is indicated when the point representing the concentrations of concurrently administered AgNP and CDDP required to achieve 50 percent loss of viability is below the line intersecting IC_50_ of AgNP and CDDP as single agents. We found that AgNP in combination with CDDP showed synergy in both A2780 and OVCAR3 cells (Figures 5(d) and 5(e)). In contrast, the additive isobole showed slight antagonism (combination point above the line intersecting IC_50_ of AgNP and CDDP as single agents) in SKOV3 cells (Figure 5(f)). The combination index (CI) relative to *F*_a_ was also assessed using the algorithms established by the Chou-Talalay method [34]. A CI < 1 denotes a synergistic interaction, aCI > 1 represents antagonism, and a CI = 1 represents an additive interaction. CI is most clinically relevant with higher fractions affected (*F*_a_ > 0.5) [34]. In agreement with the additive isoboles, plotting the combination index (CI) relative to *F*_a_ of AgNP and CDDP also showed synergism (CI < 1) in A2780 and OVCAR3 cells but not in SKOV3 cells which again showed evidence of slight antagonism (CI > 1) (Figures 5(g)–5(i)).

Another aspect of Chou-Talalay analysis is the ability to calculate the dose reduction index (DRI) [34]. The DRI in our case indicates the fold by which the dosage of CDDP or AgNP may be reduced when used in combination relative to the individual agent needed for an equivalent effect; therefore, a DRI > 1 represents a favorable dose reduction index. Importantly, combining AgNPs and CDDP showed a DRI > 1 at all *F*_a_ > 0.5 in all cell lines tested for both agents (Figures 5(j)–5(l)). These data suggest that combining AgNP and CDDP may be more beneficial than each individual agent

## 4. Discussion

In this report, we examined the efficacy of AgNPs alone and in combination with CDDP for the treatment of ovarian cancer. Our results indicate that the relative sensitivity of ovarian cancer cell lines to AgNPs is heterogeneous. AgNPs were highly cytotoxic toward A2780 and SKOV3 cells, but OVCAR3 cells were less sensitive to AgNPs. Conversely, CDDP cytotoxicity was similar across all cell lines tested. In agreement with the cytotoxicity data, AgNPs caused DNA damage in A2780 and SKOV3 cells, but not in OVCAR3 cells. Notably, for AgNP-sensitive cell lines, only 6 h exposure to AgNPs was needed to induce significant toxicity. Additionally, the most sensitive cell lines tested harbored the highest basal ROS levels. The combination of AgNPs and CDDP was synergistic for treatment of A2780 and OVCAR3 cells but was not synergistic (though nearly additive) for treatment of SKOV3 cells. Importantly, the combination of AgNPs and CDDP showed dose reducing capabilities in all cell lines tested. These results provide insight into potential applications of AgNPs for treatment of ovarian cancer alone and in conjunction with CDDP.

AgNP cytotoxicity is dependent on a variety of factors such as the types of cells exposed and also nanoparticle characteristics (size, shape, capping agent, and surface charge) [35]. However, the heterogeneity between various laboratory preparations of AgNPs and potential contamination of AgNPs with Ag+ makes comparative analysis among published studies difficult. The availability and use of high quality commercial formulations of AgNPs, including the particles used in these studies, is now enabling researchers to build upon previous results. Similar PVP-coated AgNPs to those used in this study show very little silver ion release (0.005% by mass) after 24 hours in biological conditions [36]. In agreement with this previous study [25], we found that any dissolution products released during extended AgNP storage in physiologic buffer do not induce cytotoxicity. Additionally, both OVCAR3 and A2780 cells are similarly sensitive to Ag+ exposure but showed heterogeneous sensitivity to AgNP. We further showed that endocytosis of the AgNPs is required, at least in part, for the cytotoxic effects noted. Thus, the cytotoxicity we observed was due to the nanoparticle formulation itself and not due to particle dissolution and Ag+ release prior to cell uptake.

The cytotoxicity of AgNPs was previously investigated by others in individual ovarian cancer cell lines [22, 23]. However, prior to our study, the effects of AgNPs in multiple ovarian cancer cell lines had not been examined, nor had AgNP-induced DNA damage been quantified in ovarian cancer cells. All three cell lines we tested are generally used as models for high-grade serous carcinoma, though there is some debate on whether these may be further stratified into different molecular subtypes based on mRNA expression profiles [37]. Both OVCAR3 and SKOV3 cells possess loss-of-function mutations in *TP53* [38], but A2780 cells are *TP53* wild-type [39]. Furthermore, loss of p53 activity is associated with CDDP resistance and decreased survival in ovarian cancer patients [40]. Because both SKOV3 and A2780 cells are sensitive to AgNPs while OVCAR3 cells are insensitive, the results suggest that p53 status alone does not differentiate AgNP sensitivity across ovarian cancer cell lines. This is supported by recent studies in which AgNPs induce p53-independent apoptosis in sarcoma cell lines [41]. Interestingly, we found that p53 status had no correlation with CDDP sensitivity, as p53-mutated OVCAR3 were equally sensitive to CDDP (Figure 1). However, it is important to note that resistance to CDDP is a multifactorial process [42] that may not be well reflected in the models used in this study. Evidence that p53 status alone does not impact AgNP sensitivity demonstrates that AgNPs may be more advantageous for use in a clinically relevant patient population and should be evaluated further.

Ovarian cancers frequently (~12%) exhibit inactivating defects in DNA mismatch repair (MMR) [43] and hereditary ovarian cancers often present with BRCA mutations resulting in impaired repair of DNA double strand breaks [44, 45]. Interestingly, while the most sensitive cell lines (A2780 and SKOV3) have intact BRCA genes, they both show defects in MMR. Conversely, OVCAR3 cells have both intact BRCA genes and an intact MMR [46]. Our data shows that the most sensitive cell lines (SKOV3 and A2780) showed AgNP-induced DNA damage but the less sensitive cell lines (OVCAR3) did not show DNA damage. This suggests that cancers harboring defects in DNA repair may be the most sensitive to AgNP-based therapy. Further studies will be needed to determine whether indeed there are molecular subtype-specific differences and DNA repair pathway defects that may denote tumor-specific AgNP sensitivities.

We also noted that the cell lines with the highest levels of basal ROS (A2780 and SKOV3) were the most sensitive to AgNP. We previously demonstrated that treatment with various antioxidants can reduce AgNP toxicity and that depletion of glutathione using BSO sensitizes cells to AgNPs [25]. Moreover, it is widely believed that AgNPs act as a “Trojan horse” to deliver Ag metal across cell membranes, but degradation of AgNPs into Ag+ is needed for cytotoxicity [47]. It is possible that high basal ROS liberates Ag+ for AgNPs [48] and that this contributes to the differences in sensitivity to AgNPs among the cell lines we tested.

Currently, CDDP is used as part of the standard of care for ovarian cancer, but its use is hampered by severe, dose-limiting, toxic side effects [29]. When we combined CDDP and AgNPs, we found synergism in two (A2780 and OVCAR3) of the three cell lines tested. Treatment of the third cell line (SKOV3) with CDDP and AgNPs was essentially additive. Interestingly, a high degree of sensitivity to AgNPs was not required for the synergy noted between AgNPs and CDDP as we found synergistic interactions in the less sensitive cell line OVCAR3 as well as the more sensitive cell line A2780. This evidence suggests that the underlying mechanism of synergy is not simply due to AgNP sensitivity as a single agent. Because AgNPs are known to induce a wide range of effects in cells including cell cycle arrest, inflammatory signaling (e.g., nitric oxide secretion, TNF*α* activation), and alterations in oxidative response [47], further studies are required to elucidate the specific mechanism of synergy we found when AgNPs are used in conjunction with CDDP.

Importantly, in all cell lines tested, including the less sensitive cell line OVCAR3, combining AgNPs and CDDP resulted in a DRI > 1 at relevant *F*_a_ (>0.5), showing the potential to use a decreased dose of CDDP in conjunction with AgNPs which may decrease toxic side effects in future studies. The synergistic interaction and dose reducing capabilities of AgNPs combined with CDDP may have important ramifications if AgNPs progress through preclinical studies and into the clinic with regard to improved dosing regimens and reduced off-target effects.

While full in vivo toxicity profiles have not yet been performed for our AgNPs, studies in rodents using AgNPs similar to those used by us show that AgNP toxicity is manageable at doses up to 6mg/kg following repeated, daily intravenous injections for 28 days [49] and that AgNPs do not affect platelet aggregation, coagulation, or complement activation [50]. This suggests that there is a window for the safe use of AgNPs in vivo. However, detailed in vivo studies will be needed to fully evaluate the efficacy and safety of combined AgNP and CDDP chemotherapy.

Treatment of cancer is complex and individualized approaches may be needed for each patient, depending upon the type of disease. Here, we provide evidence that a subset of ovarian cancer cell lines is particularly sensitive to AgNP therapy, both alone and in conjunction with standard-of-care platinum-based therapy CDDP. This combination has the ability to reduce required chemotherapy doses and may improve efficacy. Based on our findings, further studies are warranted to develop AgNPs as a cytotoxic agent alone and in conjunction with standard-of-care therapies and to define which subsets of ovarian cancer are best treated with AgNPs.

## Supplementary Material

Supplementary Fig S1

Supplementary Table 1

## Abbreviations

AgNP: Silver nanoparticle
BSO: Buthionine sulfoximine
CDDP: Cisplatin, *cis*-diamminedichloroplatinum(II)
Cyto D: Cytochalasin D
DLS: Dynamic light scattering
DNA: Deoxyribonucleic acid
DRI: Dose reduction index
ELISA: Enzyme-linked immunosorbent assay
*F*_a_: Fraction affected
FBS: Fetal bovine serum
GSH: Glutathione
GSSG: Glutathione disulfide
H2DCF-DA: 2′-7′-Dichlorodihydrofluorescein diacetate
MMR: Mismatch repair
MTT: 3-(4,5-Dimethylthiazol-2-yl)-2,5-diphenyltetrazolium bromide
PBS: Phosphate-buffered saline
PVP: Polyvinylpyrrolidone
ROS: Reactive oxygen species
TEM: Transmission electron microscopy
