# Supplementary material for: Heterogeneous Responses of Ovarian Cancer Cells to Silver Nanoparticles as a Single Agent and in Combination with Cisplatin
Source: J Nanomater. Author manuscript; Available in PMC 2018 Jul 19. (PMC6052800; doi:10.1155/2017/5107485)
Supplement: Supplementary Fig S1 [file NIHMS942300-supplement-Supplementary_Fig_S1.pptx]

## Slide 1
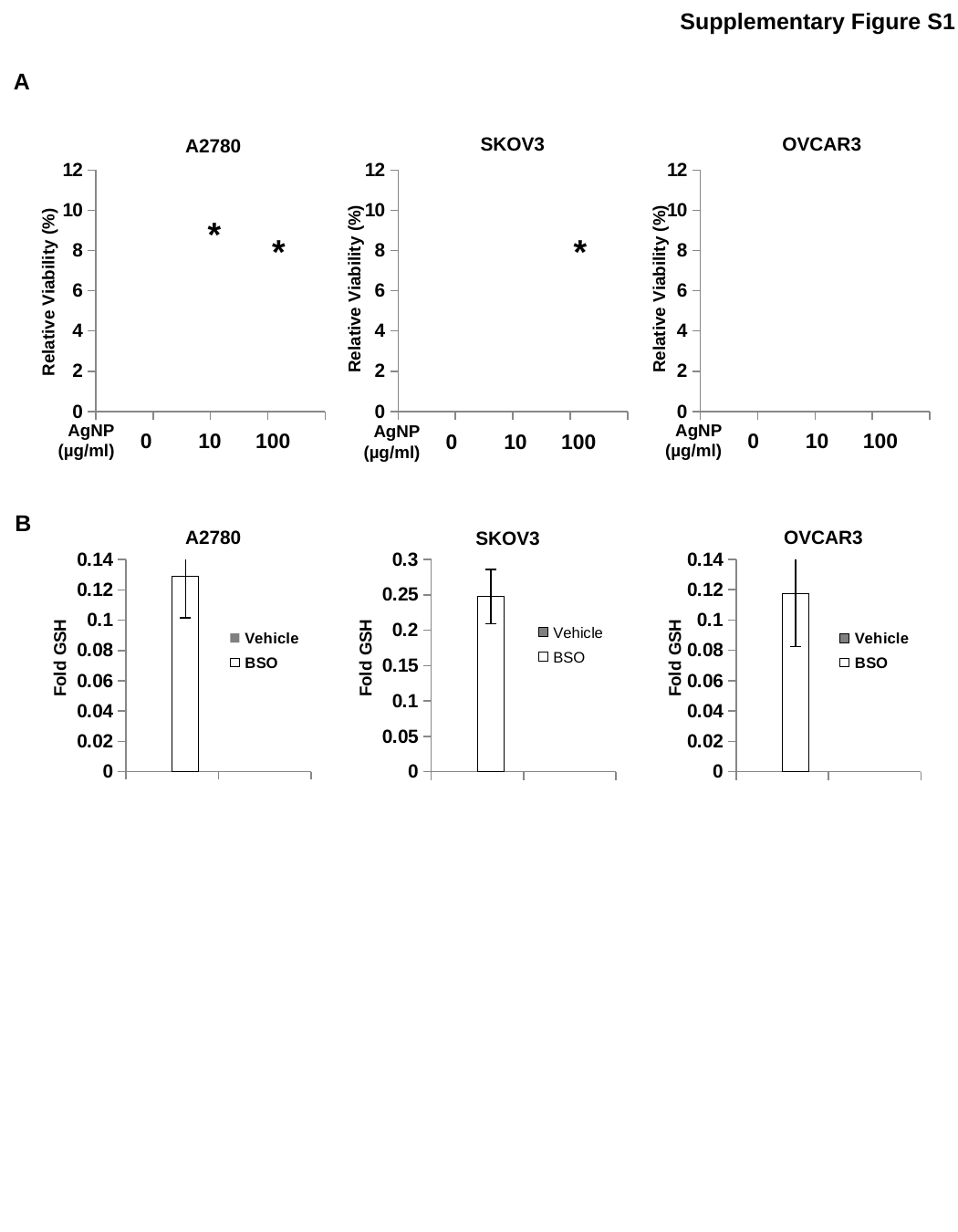

Supplementary Figure S1
A
SKOV3
OVCAR3
A2780
### Chart
| Category | Percent |
|---|---|
| Veh | 100.0 |
| 10 µg/ml AgNP | 76.25154926598016 |
| 100 µg/ml AgNP | 64.68270219107568 |
### Chart
| Category | Percent |
|---|---|
| Veh | 100.0 |
| 10 µg/ml AgNP | 95.30655004561729 |
| 100 µg/ml AgNP | 71.06371307938154 |
### Chart
| Category | Percent |
|---|---|
| Veh | 100.0 |
| 10 µg/ml AgNP | 91.77756106731209 |
| 100 µg/ml AgNP | 92.10231344949537 |
*
*
*
Relative Viability (%)
Relative Viability (%)
Relative Viability (%)
AgNP
(µg/ml)
0
10
100
AgNP
 (µg/ml)
0
10
100
AgNP
 (µg/ml)
0
10
100
B
A2780
OVCAR3
SKOV3
### Chart
| Category | Vehicle | BSO |
|---|---|---|
| Total | 1.0 | 0.2473430701127842 |
### Chart
| Category | Vehicle | BSO |
|---|---|---|
| Total | 1.0 | 0.1174276557048597 |
### Chart
| Category | Vehicle | BSO |
|---|---|---|
| Total | 1.0 | 0.12901158781471012 |Fold GSH
Fold GSH
Fold GSH
