# Supplementary material for: Heterogeneous Responses of Ovarian Cancer Cells to Silver Nanoparticles as a Single Agent and in Combination with Cisplatin
Source: J Nanomater. Author manuscript; Available in PMC 2018 Jul 19. (PMC6052800; doi:10.1155/2017/5107485)
Supplement: Supplementary Table 1 [file NIHMS942300-supplement-Supplementary_Table_1.pptx]

## Slide 1
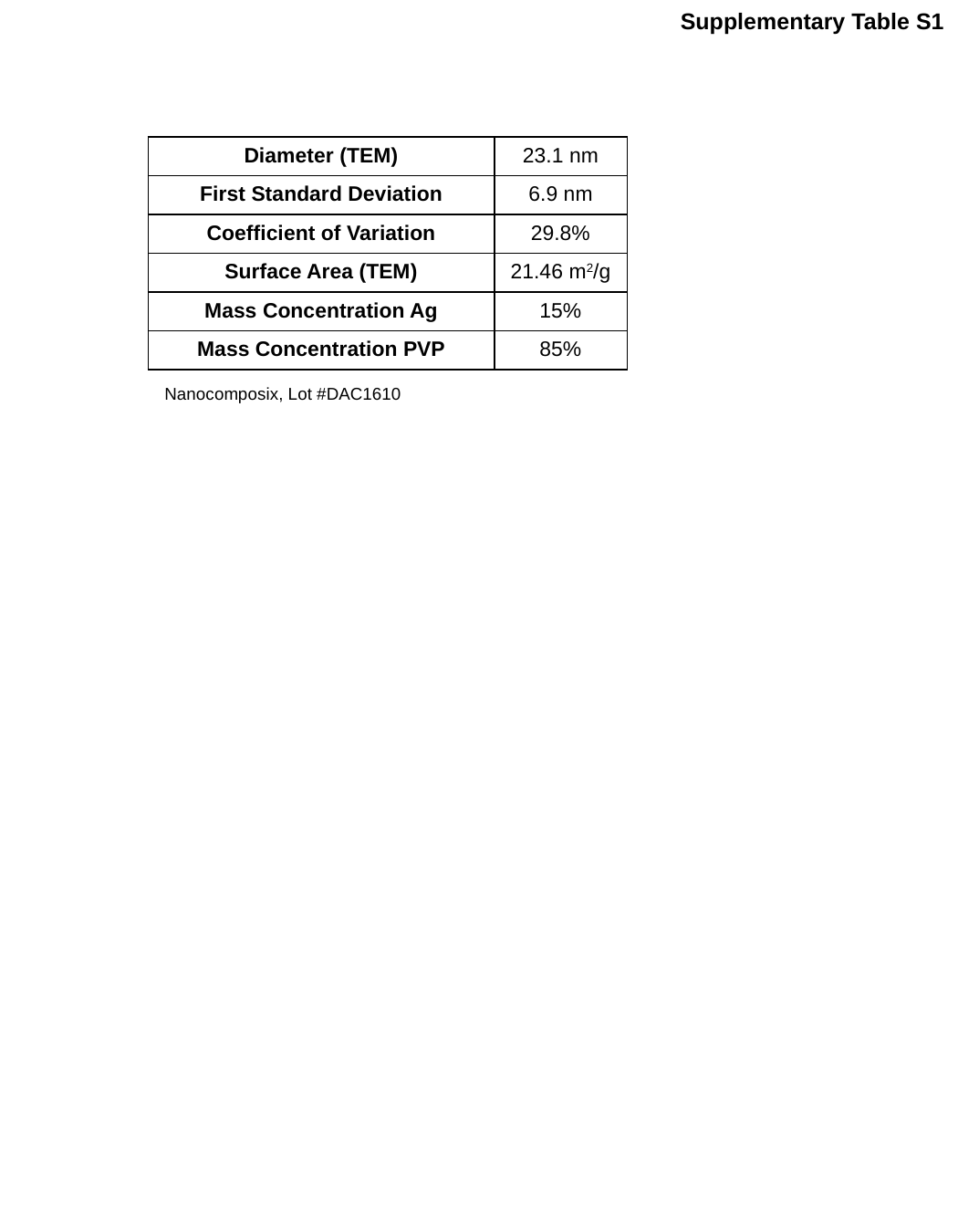

Supplementary Table S1
| Diameter (TEM) | 23.1 nm |
| --- | --- |
| First Standard Deviation | 6.9 nm |
| Coefficient of Variation | 29.8% |
| Surface Area (TEM) | 21.46 m2/g |
| Mass Concentration Ag | 15% |
| Mass Concentration PVP | 85% |
Nanocomposix, Lot #DAC1610
